# Editorial: Emerging methodologies in genotype-phenotype models for crop improvement

**DOI:** 10.3389/fpls.2025.1758547

**Published:** 2025-12-18

**Authors:** Gerhard Buck-Sorlin, Lifeng Xu

**Affiliations:** 1 Univ Angers, Institut Agro, INRAE, IRHS, SFR QUASAV, Angers, France; 2 Department of Computer Science & Technology, Zhejiang University of Technology, Hangzhou, Zhejiang, China

**Keywords:** breeding, genotype, method, modelling, phenotype

The Research Topic on genotype-phenotype models (GPM) of crops aimed at compiling articles contributing to advance our understanding of the intricate relationship between genetic information and (eco)physiological processes in plants. We are pleased to ascertain that this Research Topic has fulfilled its objectives by successfully gathering seven diverse contributions (**Table 1**), reflecting a variety of approaches, disciplines, and hierarchical scales at which the models were implemented.

**Table 1:** Classification of the seven studies featured in the Research Topic according to the primary methodological approach and biological scale.

Biological scale	Model type	
	Mechanistic	Data-driven/statistical
Molecular/Metabolic	Basallo et al.	
Individual/Organ	Lu et al.	Powadi et al.
Population/Field	Lang et al., Jin et al.	Argaw et al.
Community/System	Wolff et al.	

Lu et al. presented a novel analytical framework called Differential Interaction Regulatory Equations (DIRE) to map the genetic architecture underlying trait covariation in *Populus euphratica*, a desert tree species. The researchers developed mathematical models based on Lotka-Volterra equations to describe cooperation-competition patterns between paired traits and integrated these into QTL mapping to identify genetic loci regulating trait interactions, detecting 93–94 significant QTLs.

Lang et al. presented a revised process-based phenology model (TPForc) that incorporates photoperiod and temperature triggers for bud growth initiation in winter deciduous forest trees across China’s monsoon region. Analysing phenological data from four tree species at 102 stations, they found that photoperiod lengthening initiated bud growth in 80.8% of leaf unfolding and 77.7% of flowering time series, with a clear north-south gradient where photoperiod dependence increases toward lower latitudes.

Wolff et al. used a spatially-explicit individual-based model to investigate how intraspecific genetic variation (IV) affects interspecific competition in binary forage mixtures. They simulated 63 virtual plant communities with varying levels of functional divergence between species for light and nitrogen acquisition. The model predicted that moderate to high IV levels improved species balance maintenance over time without affecting mixture productivity, with the stabilizing effect strongest under low nitrogen conditions.

Jin et al. developed a spatiotemporal light interception model for soybean-maize strip intercropping systems using field experiments across two sites in China. The model showed high accuracy (R² > 0.814 for photosynthetically active radiation) and indicated that increasing soybean rows enhanced light interception for soybean and lower maize layers but decreased it for upper maize layers.

Basallo et al. presented a comprehensive mathematical model of the mevalonic acid (MVA) and methyleritritol-4-phosphate (MEP) pathways in plants, investigating how circadian rhythms affect the biosynthesis of terpenoid precursors IPP and DMAPP. The authors developed ordinary differential equation models incorporating three levels of circadian regulation: substrate availability, gene expression, and product utilization. Despite significant seasonal and latitudinal variations in daylight hours, IPP and DMAPP concentration oscillations remain remarkably stable, highlighting the robustness of these biosynthetic systems.

Argaw et al. presented comprehensive analysis of multi-environment trials (MET) data using linear mixed model-based approaches, comparing traditional randomized complete block (RCB) designs with spatial analysis and spatial + genotype-by-environment (G×E) analysis methods. Using ten MET grain yield datasets from Ethiopian national variety trials, the authors demonstrated that incorporating spatial variability modelling and factor analytic (FA) models for G×E effects substantially improves genetic parameter estimates and heritability calculations.

Powadi et al. presented a deep learning approach using Compositional Autoencoders (CAE) to improve maize yield prediction from high-resolution plot-level satellite imagery by disentangling genotype and environment features. Analysing approximately 4,000 satellite images from 84 maize hybrids across five U.S. Corn Belt locations, CAE-based features improved early-stage yield predictions by up to 10% compared to traditional autoencoder features and outperformed vegetation indices by 9%, demonstrating strong generalizability across different growth stages.

## Advancing genotype-phenotype models: integrating complexity across scales

These seven studies collectively advance genotype-phenotype modelling by addressing critical limitations in existing approaches that typically examine single traits or genotypes in isolation under static conditions.

A central theme is the integration of dynamic environmental interactions with genetic mechanisms. Lu et al., Lang et al., Jin et al., and Powadi et al. demonstrate how environmental factors—including resource competition, photoperiod, temperature thresholds, light dynamics, and satellite-derived environmental signals—interact with genetic traits to determine phenotypic outcomes. These studies move beyond static models to capture temporal and spatial variations in plant responses throughout development.

Several articles emphasize modelling trait interactions and emergent properties. Lu et al. explicitly model genetic covariation between traits, while Wolff et al. show how within-species genetic diversity creates community-level emergent properties through differential competitive abilities. Powadi et al. demonstrate how disentangling genotype and environment effects through deep learning enables more accurate prediction of G×E interactions. This shift from single-trait to multi-trait and multi-scale perspectives represents a fundamental advancement in capturing biological system complexity.

Mechanistic modelling of regulatory networks constitutes another common thread. Basallo et al. provide detailed mathematical models linking circadian clock genes to metabolic pathway regulation, demonstrating how genetic information controlling enzyme expression translates into predictable biochemical phenotypes.

Statistical and analytical methodology improvements appear across multiple studies. Argaw et al. demonstrate sophisticated variance structure modelling using factor analytic approaches, while Powadi et al. employ compositional autoencoders for feature disentanglement. Other articles use individual-based modelling (Wolff et al.) and functional-structural plant modelling (Lu et al., Jin et al.) to better capture genetic effects under varying conditions.

Collectively, these works enhance GPM research by integrating genetic architecture, environmental dynamics, trait interactions, and multi-scale processes, improving predictions of plant performance and accelerating breeding efficiency.

